# Serum CD26 is related to histopathological polyp traits and behaves as a marker for colorectal cancer and advanced adenomas

**DOI:** 10.1186/1471-2407-10-333

**Published:** 2010-06-28

**Authors:** Loretta De Chiara, Ana M Rodríguez-Piñeiro, Francisco J Rodríguez-Berrocal, Oscar J Cordero, David Martínez-Ares, María Páez de la Cadena

**Affiliations:** 1 Universidad de Vigo. Facultad de Biología. Departamento de Bioquímica, Genética e Inmunología. As Lagoas-Marcosende s/n. 36310 Vigo, Spain; 2 Universidad de Santiago de Compostela. Departamento de Bioquímica y Biología Molecular. Edificio CIBUS, Campus Sur. 15782 Santiago de Compostela, Spain; 3 Complejo Hospitalario Universitario de Vigo. Servicio de Digestivo. Calle Pizarro 22. 36204 Vigo, Spain

## Abstract

**Background:**

Serum CD26 (sCD26) levels were previously found diminished in colorectal cancer (CRC) patients compared to healthy donors, suggesting its potential utility for early diagnosis. Therefore we aimed to estimate the utility of the sCD26 as a biomarker for CRC and advanced adenomas in a high-risk group of patients. The relationship of this molecule with polyp characteristics was also addressed.

**Methods:**

sCD26 levels were measured by ELISA in 299 symptomatic and asymptomatic patients who had undergone a colonoscopy. Patients were diagnosed as having no colorectal pathology, non-inflammatory or inflammatory bowel disease, polyps (hyperplastic, non-advanced and advanced adenomas) or CRC.

**Results:**

At a 460 ng/mL cut-off, the sCD26 has a sensitivity and specificity of 81.8% (95% CI, 64.5-93.0%) and 72.3% (95% CI, 65.0-77.2%) for CRC regarding no or benign colorectal pathology. Clinicopathological analysis of polyps showed a relationship between the sCD26 and the grade of dysplasia and the presence of advanced adenomas. Hence, a 58.0% (95% CI, 46.5-68.9%) sensitivity detecting CRC and advanced adenomas was obtained, with a specificity of 75.5% (95% CI, 68.5-81.0%).

**Conclusions:**

Our preliminary results show that measurement of the sCD26 is a non-invasive and reasonably sensitive assay, which could be combined with others such as the faecal occult blood test for the early diagnosis and screening of CRC and advanced adenomas. Additional comparative studies in average-risk populations are necessary.

## Background

Colorectal cancer (CRC) is one of the four most prevalent cancers in Western countries due to a low recovery rate in advanced stages, when micrometastases may be already present. It develops from precancerous lesions (adenomas) that are easily removed by polypectomy, a procedure that reduces CRC incidence by 75-90% [1]; moreover, treatment of early diagnosed tumours has a good prognosis [2]. Furthermore, according to the MISCAN-COLON simulation model, a 23% reduction in CRC mortality would be achieved with the screening of at least 70% of the target population [3]. Therefore screening for CRC aims to reduce mortality rates by detection and removal of early-stage tumours, and incidence rates by identification and resection of polyps [4].

There is a great variety of methods for the detection of CRC. A review of the ongoing screening initiatives worldwide was recently published [5]. The latest Joint Colorectal Cancer Screening Guidelines [6] divide the available tests into two categories: those primarily aimed to detect cancer (blood and DNA stool tests), and those directed to detect also advanced lesions (endoscopic and radiographic methods).

Within the available invasive tests are the flexible sigmoidoscopy, the double-contrast barium enema and the colonoscopy. The latter is recommended nowadays as the gold standard, though its miss rates have been estimated as 22% for adenomas and 2-6% for CRC [7]. However, bowel preparation constitutes the most objectionable aspect of the procedure, fundamental for a proper screening colonoscopy [8]. Other limitations of the colonoscopy are the risk of complications, the costs, and access. Regarding non-invasive tests, the most common method, nowadays recommended for CRC screening, is the guaiac faecal occult blood test (gFOBT), with highly variable and brand-dependent sensitivities and specificities [6,9] and requiring dietary restrictions.

Thus there is an imperative need for developing non-invasive screening tests for the detection of CRC and adenomas, and hence many current studies are evaluating serum markers. Examples of these are the α-defensins [10], the nicotinamide N-methyltransferase [11], the α-L-fucosidase [12], or the colon cancer-specific antigens (CCSA) -2, -3 and -4 [13,14]. A major drawback in these studies is they are limited to discriminating between CRC patients and healthy individuals, leaving aside precursor lesions and not including in the control cohort individuals with benign pathologies.

Previous studies of our group detected that soluble serum CD26 (sCD26) levels were diminished in CRC patients as compared to healthy donors [15,16]. In those studies, a sCD26 cut-off of 410 ng/mL demonstrated a 90% diagnostic efficiency, with a specificity and sensitivity of 90% as well [15]. This high diagnostic efficiency, even in early tumour stages, suggested its potential utility for diagnosis. Thus we considered of interest to extend the validation of the sCD26 as an early biomarker for CRC, including also precancerous lesions (adenomas). One of the novelties of the study is the use of a colonoscopically healthy cohort instead of a blood donor cohort as the control population. Moreover, this is the first time the sCD26 is analyzed regarding the clinicopathological characteristics of colorectal polyps. Thus, we aim to analyse the relationship of the serum CD26 levels with the colonoscopic findings, in order to validate the utility of this protein as a marker for CRC diagnosis.

## Methods

### Population

The population studied consisted of patients from the CHUVI hospital (Complejo Hospitalario Universitario de Vigo; Spain) who had undergone colonoscopy at the Gastroenterology Department. All the procedures described were performed according to the clinical ethical practices of the Spanish Government. The study was approved by the Galician Ethical Committee for Clinical Research (2002/059) and complied with the tenets of the Helsinki Declaration, the Oviedo Agreement, the Organic Law for Data Protection 15/1999 and the Royal Decree 1720/2007. Anonymity was warranted through the use of clinical history numbers. For patients willing to be included in the study, informed consent was obtained and blood was extracted. The only exclusion criterion was a non-completed colonoscopy (understood as completed when the caecum was reached). The 299 patients included were both males and females, with ages ranging from 18 to 93 years. Clinical histories were recorded, including symptoms of bowel disease, personal history of polyps, CRC and other cancers, family history of cancer, and colonoscopy indication. This was mostly due to symptoms as rectal bleeding (35.5%), abdominal pain (10%), diarrhoea (9%), constipation (5.7%), anaemia (5%), but also for colorectal polyp (12.7%) or cancer (4.3%) surveillance, or CRC screening (12.4%). All procedures were blinded.

Based on the histological report, patients were classified into: no colorectal pathology, non-inflammatory bowel disease (non-IBD), inflammatory bowel disease (IBD), polyps and CRC; polyps were sub-classified as hyperplastic or adenomas. Advanced adenomas were defined as those larger than 10 mm, with tubulovillous or villous histology, or with high-grade dysplasia. Patients with more than one polyp were classified according to the most advanced lesion. CRC patients were classified according to Dukes' staging. No carcinomas *in situ *were detected in the patients included in the study.

### Collection of blood samples and determination of the sCD26 levels

Blood samples were collected, coagulated at room temperature for 20 min, and centrifuged at 2,000 *g *15 min. Sera were stored at -80°C. The sCD26 concentration was measured with the sCD26 ELISA kit (Bender Medsystems; Vienna, Austria) according to the manufacturer's instructions. Colorimetric quantification was performed with a microplate reader (model 550; Bio-Rad, USA) at 450/570 nm.

### Statistical analysis

Statistical analyses were performed with the SPSS package (v16.0); tests were two-sided; *p*-values < 0.05 were considered significant. Normal distributions and homogeneity of variances were verified by Kolmogorov-Smirnov and Levene';s tests, respectively. Analysis of two independent samples was done by Mann-Whitney's *U*, whereas for more samples we employed the Kruskal-Wallis test. Chi-square or Fischer's exact tests were done with contingency tables, and applied for analyses regarding positivity/negativity of the sCD26. Bonferroni, false discovery rate (FDR) and SGoF tests were subsequently performed with the SGoF metatest software to correct for multiple comparison [17]. The sCD26 ability to separate healthy from diseased patients was studied by receiver operating characteristic (ROC) curves. Sensitivity and specificity were calculated using MedCalc (v.10.0.2).

## Results

### sCD26 levels according to the colonoscopic results

According to the colonoscopic diagnosis, the 299 patients were classified as 68 patients with no colorectal pathology (symptomatic with rectal bleeding, abdominal pain, diarrhoea, anaemia, constipation, or asymptomatic with personal history of polyps or CRC, and family history of polyps); 64 patients with non-IBD (haemorrhoids and diverticula); 26 patients with IBD (colitis or Crohn's disease); 108 patients with colorectal polyps (hyperplastic polyps, non-advanced adenomas and advanced adenomas); and 33 patients with CRC.

As shown in table 1, the average sCD26 level for the 6 groups with no colorectal pathology was 641.2 ± 241.2 ng/mL; individuals with anaemia showed a considerably low sCD26 mean (370.8 ± 144.7 ng/mL), statistically different from other non-colorectal pathology patients (*U *test *p *= 0.001). Regarding patients with colorectal pathology, the non-IBD group exhibited mean sCD26 levels similar to the non-colorectal pathology group (612.2 ± 231.0 ng/mL). In contrast, patients with IBD showed lower values with relatively more variation (434.4 ± 239.7 ng/mL). Patients diagnosed as having polyps (both hyperplastic and adenomas) resulted in an intermediate mean sCD26 concentration (588.0 ± 246.5 ng/mL), while patients with CRC registered the lowest levels (403.7 ± 278.2 ng/mL).

**Table 1:** Average sCD26 concentration for the groups studied according to the colonoscopy result.

No colorectal pathology	Clinical condition	*n*	Mean ± SD sCD26 (ng/mL)	
	Rectal bleeding	8	651.5 ± 190.2	
		
	Abdominal pain	10	586.1 ± 194.4	
		
	Diarrhoea	9	649.5 ± 306.8	
				641.2 ± 241.2
	Anaemia	7	370.8 ± 144.7	
		
	Constipation	4	782.7 ± 194.6	
		
	No symptoms*	30	698.5 ± 234.2	

**Colorectal ** **pathology**	**Diagnosis**	***n***	**Mean ± SD** **sCD26 (ng/mL)**	

***Non-inflammatory***	Haemorrhoids	36	655.3 ± 240.4	
***bowel disease***				612.2 ± 231.0
***(non-IBD)***	Diverticula	28	556.7 ± 209.6	

***Inflammatory ***	Colitis	20	413.2 ± 195.5	
***bowel disease***				434.4 ± 239.7
***(IBD)***	Crohn's disease	6	505.1 ± 355.8	

	Hyperplastic polyps	18	560.4 ± 201.3	
		
***Polyps***	Non-advanced adenomas	40	611.1 ± 263.8	588.0 ± 246.5
		
	Advanced adenomas	48	566.7 ± 225.8	

	Duke's A	2	513.9 ± 240.4	
		
	Duke's B	12	369.8 ± 147.4	
***CRC***				403.7 ± 278.2
	Duke's C	15	402.0 ± 360.3	
		
	Duke's D	4	457.0 ± 323.0	

SD: standard deviation of the mean; *: personal history of polyps or CRC, or familial history of CRC.

The Kolmogorov-Smirnov test demonstrated, except for the CRC population (*p *= 0.035), a normal distribution of the sCD26 concentration for all the groups. When the levels of sCD26 were compared among all the five groups of patients, we found significant differences (Kruskal-Wallis test *p *< 0.001). Moreover, this differences were not related to gender (*U *test *p *= 0.219) or age (≤ 50 years or > 50 years; *U *test *p *= 0.109), as reported in a large cohort study [18].

### ROC curve and sCD26 positivity

To evaluate the utility of the sCD26 as a tumour marker, we estimated a cut-off to differentiate the CRC group (33 patients) from the control population (68 patients with no colorectal pathology). First we constructed the ROC curve, which resulted in an area of 0.811 (95% CI, 0.721-0.882; *p *< 0.0001). On the basis of this plot, table 2 shows four different potential cut-off values with their respective sensitivity and specificity. Sensitivity for CRC almost reached 85% at the cut-off of 500 ng/mL, but with a specificity below 75%; when specificity was raised up to 83.8% at 390 ng/mL, sensitivity resulted in a 57.6%. The cut-off value with the highest average diagnostic performance was 460 ng/mL, showing a sensitivity of 81.8% (95% CI, 64.5-93.0%) with a specificity of 79.4% (95% CI, 67.9-88.3%).

**Table 2:** Sensitivity and specificity of the sCD26 at different cut-off values for separating individuals with no colorectal pathology from those with CRC.

Cut-off (ng/mL)	Sensitivity (95% CI)	Specificity (95% CI)	+LR	-LR
**390**	57.6% (38.2-74.5)	83.8% (72.9-91.6)	3.6	0.5

**410**	72.7% (54.5-86.7)	80.9% (69.5-89.4)	3.8	0.3

**460**	81.8% (64.5-93.0)	79.4% (67.9-88.3)	4.0	0.2

**500**	84.9% (68.1-94.8)	72.1% (59.9-82.3)	3.0	0.2

+LR: Positive likelihood ratio; -LR: Negative likelihood ratio.

**Figure 1:**
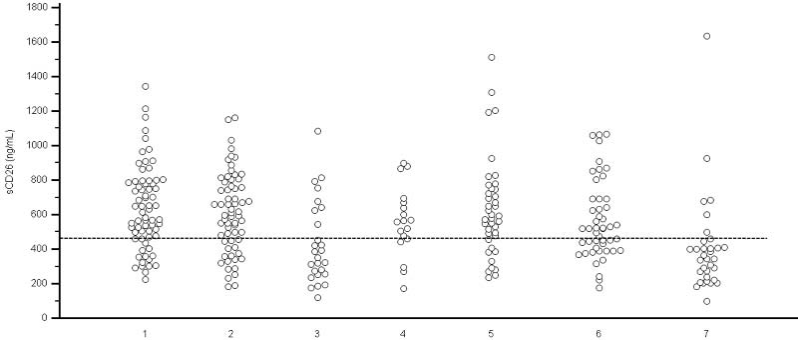
**Dot plot representing the sCD26 concentration according to the colonoscopic diagnosis**. Horizontal line: 460 ng/mL cut-off. 1: No colorectal findings (n = 68); 2: non-IBD (n = 64); 3: IBD (n = 26); 4: hyperplastic polyps (n = 18); 5: non-advanced adenomas (n = 40); 6: advanced adenomas (n = 48); 7: CRC (n = 33).

A positive sCD26 value was therefore ≤ 460 ng/mL (figure 1). The positivity rate increased from 20.6% (14/68) in the non-colorectal pathology group to 81.8% (27/33) in the CRC group. Patients with non-IBD, IBD, hyperplastic polyps, non-advanced adenomas and advanced adenomas showed intermediate rates, corresponding to 28.1% (18/64), 69.2% (18/26), 22.2% (4/18), 22.5% (9/40) and 41.7% (20/48), respectively.

### Relationship between the sCD26 and the clinicopathological features of polyps

We analysed the relationship of the sCD26 with the histopathological characteristics of the colorectal polyps found by colonoscopy (table 3), including: number of polyps (1-2, 3 or more); size (≤ 0.5 cm, 0.6-1 cm, > 1 cm); location (rectum, sigma, left colon, transverse colon, right colon); morphology (sessile, pediculated, flat); histology (hyperplasic, adenoma) and detailed histology (hyperplastic, tubular, villous, tubulovillous); grade of dysplasia (absence, low-grade, high-grade); and presence of advanced adenomas. The histology for two polyps was missing. The variables gender and age (≤ 50 and >50 years) were also studied, confirming no statistical differences in relation to the sCD26 positivity (*p = *0.212 and *p *= 0.132, respectively).

**Table 3:** Chi-square and Fischer's exact analyses for the clinicopathological characteristics of polyps in relation to the sCD26 positivity

Variable	Mean ± SD	***n*sCD26**^**+**^**/*nt***	**sCD26**^**+ **^**rate**	*p*
	sCD26 (ng/mL)			
**Number**				
1-2	577.6 ± 244.3	28/84	33.3	0.807
3 or more	624.6 ± 256.1	7/24	29.2	

**Size**				
≤ 0.5 cm	525.2 ± 208.1	11/32	34.4	
0.6 - 1 cm	673.9 ± 298.7	6/28	21.4	0.339
> 1 cm	579.9 ± 226.7	18/48	37.5	

**Location**				
rectum	549.0 ± 190.0	7/24	29.2	
sigma	617.1 ± 273.7	13/44	29.5	
left colon	571.7 ± 194.5	7/21	33.3	0.475
transverse colon	427.0 ± 157.6	4/6	66.7	
right colon	662.4 ± 324.5	4/13	30.8	

**Morphology**				
sessile	592.8 ± 264.4	18/64	28.1	
pediculated	585.3 ± 230.9	15/39	38.5	0.517
flat	548.6 ± 126.5	2/5	40.0	

**Histology**				
hyperplastic	560.4 ± 201.3	4/18	22.2	0.413
adenomas	597.2 ± 256.1	30/88	34.1	

**Detailed histology**				
hyperplastic	560.4 ± 201.3	4/18	22.2	
tubular	605.3 ± 259.9	24/72	33.3	0.358
villous	612.6 ± 305.8	2/6	33.3	
tubulovillous	529.7 ± 207.5	4/10	40.0	

**Dysplasia**				
no dysplasia	560.4 ± 201.3	4/18	22.2	
low-grade dysplasia	598.7 ± 250.5	27/83	32.5	0.056
high-grade dysplasia	562.9 ± 293.1	3/5	60.0	

**Adenomas**				
non-advanced	633.9 ± 286.9	9/40	22.5	0.048*
advanced	566.7 ± 225.8	20/48	41.7	

SD: standard deviation; *ns*CD26^+^: number of individuals with sCD26 levels ≤ 460 ng/mL; *nt*: total number of individuals per group; sCD26^+ ^rate: sCD26 positivity rate.

The Chi-square tests revealed no statistical differences regarding the number of polyps, their size, location, morphology or histology. Differences close to significant were observed between the sCD26 positivity and the grade of dysplasia (*p *= 0.056). The positivity rate increased gradually while the degree of dysplasia became more severe: 22.2% for non-dysplastic polyps, 32.5% for low-grade dysplastic adenomas and almost double (60.0%) for high-grade dysplastic adenomas. When comparing the sCD26 positivity rate of the advanced and non-advanced adenomas populations, statistically significant differences were detected (*p *= 0.048). Bonferroni, FDR and SGoF *post-hoc *tests were not significant for any of these analyses.

Finally, diagnostic parameters were calculated for the detection of advanced adenomas and CRC, with the control group including all the remaining cohorts (table 4). At the 460 ng/mL cut-off, sensitivity resulted in 58.0% (95% CI, 46.5-68.9%), with a specificity of 75.5% (95% CI, 68.5-81.0%). When these parameters were calculated for the detection of only CRC patients, the sensitivity increased to 81.8% (95% CI, 64.5-93.0%) whereas the specificity was just slightly diminished to 72.3% (95% CI, 65.0-77.2%). This value increased to 90% (95% CI, 73.4-97.8%) if the specificity was calculated for the non-symptomatic group versus the CRC group.

**Table 4:** Sensitivity and specificity for the sCD26 at the cut-off value of 460 ng/mL

Cohort	*n*sCD26^+^/*nt*	Sensitivity (95% CI)
**CRC**	27/33	81.8% (64.5-93.0)

**Advanced adenomas**	20/48	41.7% (27.6-56.8)

**CRC and advanced adenomas**	47/81	58.0% (46.5-68.9)

**Non-advanced adenomas and advanced adenomas**	30/88	34.1% (24.3-45.0)

**Cohort**	***n*sCD26^-^/*nt***	**Specificity (95% CI)**

**No colorectal findings-no symptoms**	27/30	90.0% (73.4-97.8)

**No colorectal findings**	54/68	79.4% (67.9-88.3)

**No colorectal findings, non-IBD, IBD, hyperplastic polyps, non-advanced and advanced adenomas**	191/264	72.3% (65.0-77.2)

**No colorectal findings, non-IBD, IBD, hyperplastic polyps and non-advanced adenomas**	163/216	75.5% (68.5-81.0)

*ns*CD26^+^: number of individuals with sCD26 levels ≤ 460 ng/mL; *ns*CD26^-^: number of individuals with sCD26 levels > 460 ng/mL; *nt*: total number of individuals per group.

## Discussion

The glycoprotein CD26 or dipeptidyl peptidase IV (DPPIV, E.C. 3.4.14.5) is an exopeptidase of the plasma membrane able to release dipeptides from the *N*-terminal end of peptides/proteins bearing proline or alanine in the penultimate position [19]. Biological fluids contain relatively high levels of sCD26, which is presumably shed by proteolytic cleavage from any cell expressing transmembrane CD26 [20]. Although its origin is still unclear, the liver and the T cells are cited as the most likely sources [21].

The measurement of the sCD26 levels was performed in serum from individuals whom, due to different medical indications, had undergone colonoscopy; most of them referred abdominal, colon or rectal symptoms, or familial/personal history of polyps or CCR. The individuals without colorectal findings after the colonoscopy were considered as the control cohort; the remaining were classified as: non-IBD, IBD, colorectal polyps or CRC patients. The mean sCD26 concentration decreased, although non-significantly, as the pathology diagnosed was more severe (as seen in table 1), this is, from no colorectal pathology to CRC, with a noticeable decrease in the group with IBD.

In our previous study, the control cohort was formed exclusively by healthy donors [15], whereas in the present study it was formed by individuals with confirmed no colorectal pathology but bearing symptoms, or with history of polyps or cancer. Thus we calculated a new cut-off of 460 ng/mL, higher than the value of 410 ng/mL reported for healthy donors [15].

According to this cut-off, within the no colorectal pathology group, individuals with anaemia showed a substantially elevated positivity rate (71.4%) as expected from their mean levels. Non-IBD exhibited a low positivity rate (28.1%), whereas the IBD group reached 73.1%. This pathology is associated with at least a 5-fold increased risk for CRC, representing one of the highest risk groups based on the inflammation-dysplasia-carcinoma sequence [22]. In compliance with this sequence, the sCD26 positivity rate increased from no colorectal pathology to hyperplastic polyps and non-advanced adenomas, with a further increase in advanced adenomas and CRC.

The capability of colorectal polyps to develop into cancer is related to the size of the lesion, the proportion of villous component and the grade of dysplasia. In relation to dysplasia, a morphological marker of neoplastic lesions, we observed a direct (positive) trend between the grade of dysplasia and the positivity of the biomarker, though there was no significant correlation between both parameters. Concerning advanced adenomas, a term commonly used to group adenomas that have an increased likelihood of malignant transformation, the sCD26 positivity resulted statistically significant.

Recent works also studied other potential markers in relation to polyp characteristics: for the serum sulfatase activity, differences regarding the number of adenomas (single or multiple) were significant [23]; serum leptin, adiponectin and resistin also differed between controls and patients with adenomas or CRC, though there was no relationship with dysplasia, histopathology or polyp localization [24].

In our experimental setting we have also evaluated the diagnostic parameters for the sCD26. At the 460 ng/mL cut-off, the sensitivity and specificity for CRC versus non-cancer groups were 81.8% and 72.3%, respectively. This specificity is measured in the framework of symptomatic and asymptomatic patients bearing intermediate benign pathologies including non-IBD and IBD as well as polyps. When considering only asymptomatic individuals specificity increases to 90%, which agrees with the results previously published by our group [15]; nevertheless a decrease in sensitivity will be expected in this context.

In an asymptomatic high-risk cohort of individuals with familial history of CRC or personal history of CRC or adenoma, Hazazi *et al*. [25] suggested the use of a quantitative immunochemical FOBT (iFOBT) for screening of high-risk individuals. Excluding individuals with anaemia, rectal bleeding and IBD, they reported a sensitivity and specificity for CRC of 100% and 85.3%, respectively.

In the classical CRC screening studies in average-risk individuals, the gFOBT (guaiac-based) is extensively used, despite its wide range of sensitivity and specificity. The most common tests are the Hemoccult II^® ^(rehydrated or unrehydrated) and the Hemoccult SENSA^®^, though the unrehydrated gFOBT is the one recommended for screening [26]. For the unrehydrated Hemoccult II^®^, the most accurate diagnostic parameters have been estimated with one-time testing on a Chinese [27] and an American populations [28]; in both, colonoscopy was performed to all individuals with positive or negative gFOBT results, reporting a sensitivity for CRC of 25 and 12.9%, and a specificity of 80 and 95.2%, respectively [27,28]. These studies reflect a poor sensitivity, although it is slightly improved with repeated annual or biennial testing (54-80%), reaching up to 97.7% specificity [29,30]. On the other hand, a 50% sensitivity was obtained for a one-time rehydrated testing combined with sigmoidoscopy, though no specificity was reported, perhaps owing to an increase in the number of false positives due to rehydration [31].

When a highly sensitive test like the Hemoccult SENSA^® ^is used, a 71-79% sensitivity is reached with single testing, and about 85% with multiple testing, with corresponding specificities of 86% and 95% [32,33]. However, these parameters are probably overestimated as these studies lacked colonoscopic examination of the negative cases.

Besides guaic-based FOBT, iFOBT has been recently offered as an alternative for average-risk screening. The studies reported up to date have shown that the iFOBT is more adequate for screening because of its high specificity since it detects human globin [26].

Throughout our study, evidence was gathered regarding the utility of the sCD26 in the detection of advanced adenomas. In separating CRC and advanced adenomas from all other groups, the sCD26 exhibited a 58.0% sensitivity and a 75.5% specificity. For the same pathologies (CRC and advanced adenomas) in an asymptomatic high-risk cohort, Hazazi *et al*. [25] reported for a quantitative iFOBT a sensitivity and specificity of 65.3% and 87.5%, respectively.

Regarding studies performed in average-risk individuals for the detection of CRC and advanced adenomas, gFOBT has shown a sensitivity of 10.8-14.3% [27,28] and a specificity of 79.2% [27], and consequently is not recommended for the detection of advanced lesions [4,6]. On the other hand, iFOBT showed a sensitivity of 33.1% and a specificity of 97.5%, though these parameters were given for only distal advanced neoplasm as compared to flexible sigmoidoscopy [25].

In relation to other experimental serum biomarkers for advanced adenomas, the CCSA-2 has shown a 97.3% sensitivity with a 78.4% specificity considering normal colonoscopy, hyperplastic polyps and non-advanced adenomas [13], whereas for CCSA-3 and -4 a combined sensitivity of 91.3% and a specificity of 78.7% was reported [14]. While sCD26 shows lower sensitivity, the specificity is equivalent even including patients with confounding pathologies such as IBD, which were not present in other studies.

Although sCD26 seems to perform adequately as a blood biomarker for CRC and advanced adenomas, independent of the frequent but intermittent bleeding unlike gFOBT or iFOBT, our study presents some limitations that should be considered: i) the symptomatic population included is at high-risk for CRC, with an elevated prevalence of colorectal pathology; ii) although no differences regarding age were detected, the age range of the patients differs from that recommended for screening; iii) the classification of the patients into the categories proposed resulted in several sub-groups with a small number of patients. Therefore, further research in a large population and under a screening context is desirable, along with the comparison to a sensitive gFOBT or iFOBT, and other experimental non-invasive methods.

## Conclusions

Our results show that measurement of the sCD26 is a non-invasive and reasonably sensitive assay, which could be combined with others such as the faecal occult blood test, for the early diagnosis and screening of CRC and advanced adenomas. With this aim, we are currently initiating a multicentric, prospective, double-blinded study in an average-risk population, where the performance of the quantitive iFOBT and the sCD26 assay will be assessed and compared regarding the gold standard colonoscopy.

## Competing interests

The authors declare that they have no competing interests.

## Authors' contributions

LD: measurement of the sCD26 levels, data analysis, statistical evaluation, manuscript preparation. AMRP: data analysis, statistical evaluation, manuscript preparation. FJRB: Study design, coordination of the study. OJC: data analysis, manuscript preparation. DMA: Patient recruitment, collection of samples and clinical data. MPD: Study design, coordination of the study, final revision of the manuscript. All authors read and approved the final manuscript.

## Pre-publication history

The pre-publication history for this paper can be accessed here:

http://www.biomedcentral.com/1471-2407/10/333/prepub
